# Evaluating the relationship between rental assistance and self-reliance and well-being among displaced populations: A propensity score–matched analysis

**DOI:** 10.1016/j.ssmph.2026.101948

**Published:** 2026-07-09

**Authors:** Ilana Seff, Juan Pablo Franco, Arturo Harker Roa, Deanna Barch, Kellie Leeson, Lindsay Stark

**Affiliations:** a Washington University in St. Louis, United States; b Blumont, Colombia; c Los Andes University, Colombia; d Women's Refugee Commission, United States

## Abstract

**Background:**

Housing insecurity is a central but understudied determinant of self-reliance and psychosocial well-being among forcibly displaced populations. This study evaluates the relationship between a nine-month rental assistance program on self-reliance and multiple dimensions of well-being among female-headed Venezuelan migrant households in Colombia.

**Methods:**

To construct a matched control group, 517 treated and 549 eligible untreated households were matched using one-to-one nearest-neighbor propensity score matching with replacement The average treatment effect for the treated was estimated at 2, 6, and 12 months after the intervention ended. Outcomes included overall self-reliance, peace of mind, life satisfaction, agency, self-efficacy, time availability, sense of community, acculturation, and per capita household income.

**Results:**

Matched analyses showed that rental assistance yielded statistically significant improvements across nearly all outcomes at every follow-up. At 2 months post-endline, treatment households scored 0.32–0.89 standard deviations higher than matched controls across all outcomes; except for per capita household income, these gains were sustained through 12 months post-endline. Random-effects models confirmed that early advantages persisted over time, with comparatively larger improvements observed in peace of mind and self-efficacy over time. While some treatment affects attenuated modestly for community-related outcomes, treatment households consistently outperformed controls.

**Conclusions:**

Findings provide evidence that short-term rental assistance may durably improve self-reliance and psychosocial well-being among displaced households. By alleviating housing costs, stabilizing living conditions, and enhancing perceived safety, rental assistance appears to reduce scarcity-related stress and enable longer-term planning and economic recovery. Housing support may therefore represent an efficient and impactful intervention for vulnerable displaced populations.

## Highlights

• Nine months of rental assistance led to large and sustainable gains in self-reliance and well-being for displaced households
• Effects persisted up to 12 months after rental support ended for these female-headed households
• Improvements in psychosocial well-being and self-reliance outlasted income gains.

## 1 Background

Colombia is home to approximately 2.9 million Venezuelan migrants who have fled their home country since 2014 due to a confluence of humanitarian emergencies, including economic collapse, widespread violence, and political oppression. Critically, 52% of these migrants are women and girls (Migración Colombia, 2024). Migration is a known driver of poor mental health with elevated levels of depression, anxiety, posttraumatic stress and other mental illnesses well-documented in refugee and migrant populations (Bogic et al., 2015). Female Venezuelan refugees are especially vulnerable to adverse outcomes given the complex interplay of gender and migration status that can affect their access to health services, employment, and housing (Gaviria et al., 2025). Accessibility issues, including lack of formal health insurance and high service costs, discourage Venezuelan migrants in Colombia, especially female migrants, from accessing health services (Agarwal-Harding et al., 2024). Female-headed households are also more likely to rely on informal sector employment, which disproportionately exposes them to poverty and exploitation (Jeronimo Kersh, 2021). While little research explores gender-based dimensions of housing, the United Nations recognizes that women are affected more often than men by forced evictions and other violations of the right to housing, especially in rapidly urbanizing areas, such as Colombian cities (Azcona et al., 2020).

Housing insecurity, which may comprise instability, overcrowding, poor housing quality, unaffordability, or pending eviction, has been shown to significantly compromise mental health and psychosocial outcomes. A wide body of evidence demonstrates that individuals experiencing such insecurity are more likely to report elevated levels of stress, anxiety, depression, and lower life satisfaction than those living in stable housing conditions (Roberts et al., 2025; Singh et al., 2019) More recent evidence also highlights the measurable, biological impacts of insecure housing on allostatic load, with research linking housing insecurity to increased levels of cortisol and other stress-related biomarkers (Blair et al., 2011; Clair & Hughes, 2019). These findings underscore housing insecurity as more than a material challenge, but also as a central driver of psychosocial risk and health inequality.

The mechanisms linking housing insecurity to mental well-being have been increasingly grounded in behavioral and cognitive theory. According to the scarcity mindset framework, persistent poverty and material constraints such as unstable housing can deplete cognitive bandwidth and narrow attention toward immediate survival demands (Mani et al., 2013). This cognitive tunneling can undermine the capacity for future-oriented planning, job-seeking, and saving, impeding individuals’ ability to reach economic stability, self-reliance, and well-being (Mullainathan & Shafir, 2013). As such, having access to secure, adequate, and affordable housing may not only alleviate the stress of not being able to meet a basic need, it may also free up the mental and emotional resources to support longer-term investments in health, education, and employment. Given the robust evidence on the bi-directional relationship between financial and mental well-being (Hassan et al., 2021), housing interventions may feasibly impact self-reliance through improved mental well-being, bolster well-being through heightened self-reliance, and/or effect both outcomes simultaneously. While there is some evidence that demonstrates the direct and indirect impacts of housing interventions on mental well-being, the majority of published research is limited to high-income countries that are not experiencing ongoing humanitarian crises, such as the United States (Denary et al., 2021; Fusaro et al., 2026; Jaramillo & Rohe, 2024).

In contexts of forced displacement, the urgency of housing as a determinant of well-being intensifies. Forcibly displaced households, especially those in urban settings, often live in informal or precarious housing arrangements and face affordability constraints, restricted access to formal rental markets, and elevated risk of eviction (Brown et al., 2024). These risks are even more pronounced for refugee populations, specifically, with evidence suggesting refugees exhibit the most compromised housing outcomes of all migration groups (Hiebert, 2009). At the same time, refugee populations experience disproportionately poor psychosocial and economic outcomes due to experiences before, during, and after migration (Gleeson et al., 2020). Elevated levels of depression, anxiety, posttraumatic stress and other mental illnesses are well-documented in refugee populations (Bogic et al., 2015).

As suggested above, these dual burdens create a bidirectional feedback loop for refugees: unstable housing undermines well-being and self-reliance, while weak self-reliance and mental well-being further erode housing stability. Further, despite evidence linking housing insecurity to depression and anxiety among refugees (Ziersch & Due, 2018; Seff et al., 2025 ), rigorous evaluations of housing interventions for displaced populations remain scarce. Further, in low-income contexts, the majority of individual- and household-level interventions aimed at improving the economic well-being of refugee populations tend to focus on livelihoods, with little to no robust evidence on the impact of housing interventions for refugees in these contexts (Stark et al., 2025).

The current study addresses this gap by employing propensity score matching to estimate the effects of a nine-month rental assistance program on household self-reliance and well-being among female-headed Venezuelan migrant households in Colombia. By comparing outcomes among households that received the rental support and those matched on key characteristics who did not, this analysis seeks to assess whether alleviating housing costs and improving living conditions can enhance self-reliance and lead to sustained improvements in the scarcity mindset, agency, and other outcomes of well-being.

## 2 Methods

### 2.1 Setting

We conducted this study in 17 neighborhoods in nine cities in Colombia: Barranquilla, Soledad, Cartagena, Cúcuta, Villa del Rosario, Los Patios, Cali, Jamundí, and Medellín. Colombia is home to approximately 5.8 million refugees and migrants from Venezuela, which is the largest population of Venezuelan migrants in Latin America (UNHCR, 2024; Wolf, 2021). These migrants and refugees have elevated levels of depression, anxiety and other mental health sequelae () and female-headed households are especially at risk due to limited financial security, heightened poverty, and gender-based violence exposure due to their employment in the informal sector (Jeronimo Kersh, 2021).

### 2.2 Intervention

Blumont's rental assistance program was designed to promote access to safe and adequate housing for Venezuelan migrant women and their families. Rather than being assigned housing, participants were responsible for locating a rental property that satisfied a set of minimum standards established by the program. These requirements stipulated that: (1) the landlord agree to a written lease lasting nine months; (2) no more than three people share a single sleeping space; and (3) the dwelling have reliable access to water, sanitation, and electricity. Once a household identified a potential unit, Blumont shelter officers conducted a verification visit using a structured checklist to confirm compliance with these conditions, assess privacy and safety, and ensure that the location was not exposed to significant environmental or disaster risks.

Following verification, Blumont assumed responsibility for rental payments over the nine-month period, transferring funds directly to landlords. The initial payment, which covered the first four months of rent, was made immediately after the rental agreement was signed. Subsequent payments were distributed at two-month intervals (at months four, six, and eight). On average, the program paid for monthly rents of COP $413,694 (approximately USD $103). Field teams maintained periodic contact with participant households throughout the nine months, conducting monitoring visits to ensure ongoing compliance with program standards and to identify any protection or service needs. While the intervention did not provide direct economic or psychosocial programming, staff offered referrals to local and international partners delivering social protection, livelihood, or case management support upon request.

### 2.3 Participants and procedures

Eligibility for the rental assistance program was ascertained using a standardized screening process carried out by Blumont's protection and monitoring and evaluation team. To be eligible for the housing program, participants needed to be female household heads who had migrated from Venezuela and lived in substandard or insecure housing. In October 2023, field staff conducted visits across 17 neighborhoods in nine municipalities to identify potential participants. Screening respondents were identified using snowball sampling approaches within each neighborhood. During these visits, staff completed a structured scorecard that recorded core inclusion criteria and captured additional indicators of vulnerability, such as being at-risk of gender-based violence, being pregnant or lactating, having a disability, and other protection-related risks. Households demonstrating multiple vulnerabilities or severe shelter inadequacy (e.g., residing in informal settlements, overcrowded dwellings, or structures lacking ventilation or facing imminent eviction) were assigned higher vulnerability scores.

The screening process was conducted with 1998 households and 1066 households met inclusion criteria. Resources were available to provide rental assistance for 517 households, divided approximately equally across the 17 neighborhoods. Households were ordered based on their vulnerability scores and invited to participate in the intervention beginning at the top of the list (those with higher vulnerability scores). If a household did not wish to participate in the intervention, program staff moved to the next household on the list. As a result, there was some overlap in the range of eligibility scores between treated and untreated households. At baseline, 517 and 549 households did and did not receive rental assistance, respectively.

Data for the treatment group were collected at the onset of the intervention, at endline, and two, six, and twelve months following endline. Data for those who did not receive the intervention were collected at the two-, six- and twelve-month marks only. A pre-post analysis of the treatment group data has been presented elsewhere (Stark et al., 2025). The present analysis draws on the three rounds of follow-up data collected post-endline and propensity score matching to compare outcomes for treatment and matched control households.

The data analyzed in this study were originally collected by the implementing organization as part of its routine administrative and program monitoring procedures, rather than for research purposes. As such, formal ethics approval was not sought at the time of data collection. Prior to data collection, participants were informed about the purpose of the data collection activities, the procedures involved, any potential risks and benefits, and their right to decline participation or withdraw at any time without penalty. The present study involved secondary analysis of de-identified administrative/program monitoring data and was deemed exempt from review by the Institutional Review Board at Washington University in St. Louis.

### 2.4 Outcomes of interest

The primary outcome of interest for this analysis was household-level self-reliance, measured using the Self-Reliance Index (SRI) (Seff et al., 2021). The SRI defines self-reliance as a household's ability to meet its essential economic and social needs in a stable and dignified manner, without relying on formal external assistance. The index evaluates twelve interrelated domains of self-reliance: housing quality and rent, food security, education, access to needed healthcare, health status, safety, employment, financial resources, assistance, debt, savings, and financial and informational social capital. Both domain scores and the overall score of self-reliance assume values between 1 and 5, with higher scores reflecting greater self-reliance. The SRI questions are designed as open-ended prompts rather than standardized survey designed to facilitate rapport and to enable a nuanced assessment of the household's situation. Female heads of household provided responses on behalf of all household members. The SRI was developed specifically for and validated in humanitarian and displacement contexts (Seff et al., 2021) and, to date, has been used by more than 70 refugee-serving organizations in 34 countries (Refugee Self-Reliance Initiative, 2025). The SRI has demonstrated validity among Venezuelan migrant populations in Colombia using known known-group validity testing and has been used in other program evaluations in this context (Davidson, 2023; Refugee Self-Reliance Initiative, 2021; Seff et al., 2025 ).

Several measures of various dimensions of well-being were also assessed. After examining pairwise correlations among all outcome variables (see Supplementary Table S1), it was found that correlations exceeded the conventional thresholds indicating potential redundancy in outcome variables (r ≥ 0.90) (Tabachnick & Fidell, 2019). A full list of outcomes, along with their construction and scoring, can be found in Table 1.

**Table 1:** Outcomes of well-being.

Outcome	Question(s)/Statements(s)	Scoring	Interpretation	Validity and reliability
Peace of mind (Lee et al., 2013)	1. My mind is calm, free, and peaceful 2. I feel good and comfortable with myself in everyday life 3. The way I am living makes me feel stable, calm, and peaceful 4. I feel that I have peace, calm, and harmony in my mind 5. I find it difficult to feel calm 6. The way I am living gives me feelings of comfort, peace, and calm 7. I feel anxious and mentally restless	Each item rated on 5-point Likert scale (Never to All the time); Final score is mean of the seven items	1 to 5, higher signals greater peace of mind	Developed and validated in China, including with migrant populations, but not previously validated in Spanish (Lee et al., 2013). Cronbach's alpha in the sample was 0.89 at two months post-endline.
Agency	The current conditions of my life allow me to act and/or make decisions about important goals for my life and that of my family	5-point Likert scale representing level of agreement	1 to 5, higher signals greater agency	
Life satisfaction	Understanding well-being as the satisfaction you have with your life as a whole, which step you are on today?	Five steps, higher is more satisfied	1 to 5, higher signals greater life satisfaction	
Self-efficacy	When I have a problem, I am calm and know what I need to solve it	5-point Likert scale representing level of agreement	1 to 5, higher signals greater self-efficacy	
Time availability	How would you rate the total amount of time you spend with your family, your friends, and yourself?	5-point Likert scale representing level of sufficiency	1 to 5, higher signals greater time availability	
Sense of community (McMillan and Chavis, 1986)	Four subscales (Peterson, Speer, & McMillan, 2008): Needs fulfillment: 1. I can get everything I need in this neighborhood 2. This neighborhood helps me meet my needs Membership: 3. I feel like a member of this neighborhood 4. I feel like I belong in this neighborhood Influence: 5. My opinion is valued about what happens in this neighborhood 6. People in this neighborhood help and advise each other Emotional connection: 7. I feel emotionally connected to this neighborhood 8. I have a good emotional bond with the people in this neighborhood	5-point Likert scale representing level of agreement for each item; Final score for each subscale is mean of the two underlying items	1 to 5, higher signals greater needs fulfillment, membership, influence, and emotional connection	Spanish version previously validated (Buckley et al., 2022). Cronbach's alpha for all eight items at two months post-endline was 0.90.
Acculturation	1. For me, as a migrant, it is important to preserve and share Venezuelan customs with the people of Colombia 2. For me, as a migrant, it is important to learn about and adopt Colombian customs	Scale from 1 to 10 indicating level of agreement with each statement	‘1’ if response of ≥ 8 for both items; ‘0’ otherwise	
Income	Per capita household income in previous month (COP)	Household's income in the previous month divided by household size	N/A	

### 2.5 Analysis

Given that households were not randomized to receive the housing intervention, we employed propensity score matching to identify a comparison group that closely resembled the treatment group (Rosenbaum & Rubin, 1983). Propensity score matching involves calculating each participant's probability of receiving the intervention (i.e., the propensity score) based on a set of observed baseline characteristics. Those who participated in the intervention are then matched to non-participants with similar propensity scores, thereby producing a balanced sample that minimizes bias due to observable differences between groups.

We employed psmatch2 in Stata to estimate the average treatment effect for the treated (ATT). The ATT represents the difference between the observed outcomes of households that received rental assistance and the outcomes those same households would have experienced had they not received the intervention, approximated using matched comparison households with similar characteristics at screening. The characteristics included in the model—which were found to be strongly associated with treatment status—were household income per person, household size, the respondent's age, whether the household had delays in rent payment at the time of the screening process and the neighborhood in which the household lives. Treated and untreated households were matched using one-to-one nearest-neighbor propensity score matching with replacement. Each treated household was matched to the control household with the closest estimated propensity score. Because matching was performed with replacement, some untreated households were selected as matches for multiple treated households. The resulting weights reflect the number of times each control household was used as a match, such that the weighted matched control group reproduces the distribution of observed characteristics among treated households in subsequent analyses.

We assessed the adequacy of the propensity score matching procedure by examining common support and covariate balance between treated and matched control households. Visual inspection of the propensity score distributions indicated substantial overlap between the treated and untreated households (see Supplementary file S2). The range of propensity scores among treated households (0.042–0.898; mean = 0.570) fell largely within the range observed among control households (0.020–0.865; mean = 0.443), indicating adequate common support for matching. Balance diagnostics demonstrated that the matching procedure substantially reduced baseline differences between groups. Prior to matching, treated and control households differed substantially across several characteristics, with a mean standardized bias of 27.1% (see Supplementary File S3). After matching, standardized differences across all covariates were reduced to below commonly recommended thresholds, with a mean standardized bias of 1.17%. Rubin's B and R statistics also fell within recommended ranges (B = 5.2; R = 1.04), indicating adequate balance between treated and matched control households (Stuart, 2010). These diagnostics suggest that the matching procedure successfully constructed a comparison group that closely resembled the treated households on observed baseline characteristics.

To assess differences in outcomes between the treatment and weighted matched control groups, we employed ordinary least squared regressions. Separate regression models were estimated for each outcome at each of the three rounds of follow up, with the outcome and treatment status (for the matched sample) serving as the dependent and independent variables, respectively. The matching weights were used to account for the fact that each control household may serve as a match for more than one treatment household.

To evaluate whether ATTs varied over time—i.e., whether treatment effects were stronger, weaker, or stayed the same over time—we estimated panel regression models with random effects and treatment–time interactions. Random-effects models were selected because treatment status did not vary within households across survey rounds, rendering fixed-effects estimation inappropriate for estimating the intervention effect. The random-effects specification allowed us to incorporate repeated observations for each household while accounting for unobserved, time-invariant heterogeneity across households. This approach provides a complementary assessment of the intervention's effects by examining whether treated households follow different trajectories than matched controls from 2- to 6- to 12-month follow-up. Random-effect panel regression models controlled for the variables used in the propensity score matching at screening (i.e., household income per capita, household size, the respondent's age, whether the household had delays in rent payment at the time of the screening process and the neighborhood in which the household lived) as well as the household's eligibility score during the screening process. Models were estimated using matching weights derived from the propensity score matching procedure. Because Stata does not allow clustered robust standard errors in combination with importance weights in xtreg, standard errors are based on the random-effects variance structure. All outcomes of interest were standardized prior to analysis.

## 3 Results

**Fig. 1:**
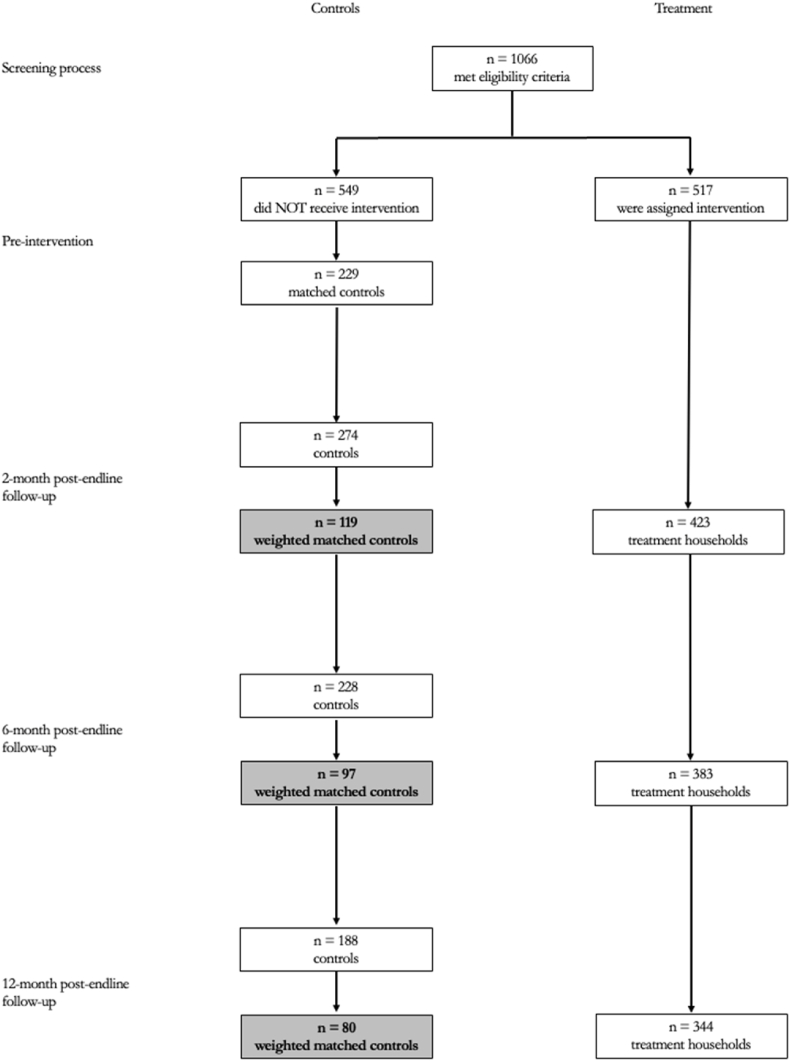
Participant flow chart.

The propensity score matching identified 229 women and their households, among those who did not receive the intervention, to serve as matched controls to the treatment households (see Fig. 1). Data were collected from 119 matched control and 423 treatment households at two months after endline, 97 matched control and 383 treatment households six months post-endline, and 80 matched control and 344 treatment households 12 months after endline. These figures reflect 18% and 48% loss-to-follow-up for treatment and matched control households, respectively, at 2 months after endline, 9% and 18% additional loss-to-follow-up at 6 months after endline, and 10% and 18% additional loss-to-follow-up at 12 months post-endline. Loss-to-follow-up was likely lower for the treatment group as Blumont field staff maintained periodic contact with treatment households during the 9-month intervention period. To assess the potential for attrition-related bias, baseline characteristics used in the propensity score matching procedure were compared between households that remained in the sample and those lost to follow-up at the first follow-up wave, separately for treatment and control groups. Differences were minimal. Specifically, among matched control households, those lost to follow-up were on average 4.7 years younger than those retained, while among treated households those lost to follow-up had household sizes that were approximately 0.39 persons smaller than those retained. No other statistically meaningful differences were observed across the remaining matching variables. Importantly, both respondent age and household size are included as covariates in the outcome regression models, which further reduces the potential influence of these differences on estimated treatment effects.

Flowchart of participants: treatment, nontreatment, and matched control households.

Summary statistics for basic demographics at screening can be found for the treatment and weighted matched control households in Table 2. The average respondent was 35.8 years of age at the screening with an average household size of 4.28. Approximately 54% of the full sample was at least one month late on rent. Mean values were statistically similar between the treatment and weighted matched control groups for all characteristics.

**Table 2:** Summary statistics of matched characteristics for treatment and weighted matched control households.

At pre-baseline screening:	Full sample		Treatment		Matched control		P-value
	(n = 746)		(n = 517)		(n = 229)		
Per capita household income (COP)	198806	[135740]	201852	[133546]	195761	[138079]	0.603
Household size	4.28	[1.50]	4.26	[1.44]	4.30	[1.56]	0.782
Respondent age	35.8	[11.43]	35.88	[11.04]	35.81	[11.66]	0.949
Venezuelan	0.98	[0.14]	0.98	[0.14]	0.98	[0.14]	0.999
At least one month late in rent	0.54	[0.50]	0.54	[0.50]	0.54	[0.50]	0.866

Note: Statistics are mean[sd]. P-values reflect tests of differences between the treatment and matched control means.

Table 3 presents the standardized ATTs at each point of data collection. Excluding acculturation and per capita household income at 12 months post-endline, all outcomes of interest were greater in the treatment group as compared to the matched control group at two, six, and twelve months after endline. Effect sizes were also large, with treatment participants generally exhibiting scores that were 0.3-0.7 standard deviations greater than weighted matched control. Particularly large effect sizes were observed for the SRI at 2 months post-endline (B = 0.89; 95% CI [0.66-1.108]; P < 0.001) and 6 months post-endline (B = 0.98; 95% CI [0.682, 1.276]; P < 0.001) and the peace of mind scale at 6 months post-endline (B = 1.04; 95% CI[0.761 - 1.310]; P < 0.001) and 12 months post-endline (B = 0.94; 95% CI[0.663 - 1.209]; P < 0.001).

**Table 3:** Standardized average treatment effects for the treated, using the matched sample.

Treatment effect:	SRI	Peace of mind	Life satisfaction	Agency	Self-efficacy	Time availability	Necessities met	Belonging	Influence	Connection	Acculturation	Per capita income
2-months after endline (n = 542)	0.89∗∗∗	0.62∗∗∗	0.42∗∗∗	0.64∗∗∗	0.32∗∗	0.35∗∗	0.54∗∗∗	0.47∗∗∗	0.61∗∗∗	0.56∗∗∗	0.65∗∗∗	0.45∗∗∗
	[0.666 - 1.108]	[0.417 - 0.828]	[0.200 - 0.648]	[0.406 - 0.870]	[0.086 - 0.562]	[0.097 - 0.597]	[0.310 - 0.762]	[0.247 - 0.688]	[0.382 - 0.837]	[0.313 - 0.806]	[0.397 - 0.895]	[0.224 - 0.677]
6 months after endline (n = 480)	0.98∗∗∗	1.04∗∗∗	0.62∗∗∗	0.65∗∗∗	0.63∗∗∗	0.33∗∗∗	0.66∗∗∗	0.60∗∗∗	0.45∗∗∗	0.37∗∗∗	0.33∗	0.56∗∗
	[0.682 - 1.276]	[0.761 - 1.310]	[0.411 - 0.830]	[0.391 - 0.914]	[0.396 - 0.866]	[0.147 - 0.517]	[0.374 - 0.938]	[0.417 - 0.791]	[0.301 - 0.608]	[0.219 - 0.512]	[0.051 - 0.614]	[0.209 - 0.916]
12 months after endline (n = 424)	0.67∗∗∗	0.94∗∗∗	0.63∗∗∗	0.68∗∗∗	0.63∗∗∗	0.63∗∗∗	0.46∗∗	0.37∗∗	0.45∗∗∗	0.36∗∗∗	0.16	0.09
	[0.429 - 0.915]	[0.663 - 1.209]	[0.364 - 0.901]	[0.367 - 0.986]	[0.434 - 0.831]	[0.431 - 0.825]	[0.156 - 0.770]	[0.147 - 0.602]	[0.221 - 0.679]	[0.182 - 0.545]	[-0.123 - 0.434]	[-0.265 - 0.447]

Note: Statistics presented are Beta coefficient and 95% confidence intervals. All effect sizes are standardized. Treatment effects are statistically significant at ∗p < 0.05, ∗∗p < 0.01, and ∗∗∗p < 0.001. All beta coefficients statistically significant at p < 0.001 are also significant at p < 0.0045, the alpha level after applying a Bonferroni correction to account for multiple comparisons.

To further examine whether treatment and matched-control households followed different trajectories over time, random-effects panel regression models with treatment–time interactions were estimated (see Table 4). Across outcomes, and as shown in both Table 3andTable 4, the main treatment coefficients indicate that intervention households scored substantially higher than matched controls at two-months post-endline. The time indicators show that control households also experienced improvements over time, with significant gains from two months after endline to both 6- and 12-month follow-ups for a few outcomes, including self-reliance, agency, and income. For example, self-reliance improved between 2- and 6-months post-endline in the matched-control group (B = 0.70; 95% CI[0.561 - 0.843]; P < 0.001), though overall levels of self-reliance were higher in the treatment group at both time points.

**Table 4:** Random-effects panel regression estimates of ATTs in the matched sample.

	SRI (n = 542)	Peace of mind (n = 542)	Life satisfaction (n = 542)	Agency (n = 542)	Self-efficacy (n = 542)	Time availability (n = 542)	Necessities met (n = 542)	Belonging (n = 542)	Influence (n = 542)	Connectio (n = 542)n	Acculturation (n = 542)	Per capita household income (n = 542)
Treatment	0.87∗∗∗	0.58∗∗∗	0.40∗∗∗	0.66∗∗∗	0.32∗∗∗	0.32∗∗∗	0.46∗∗∗	0.39∗∗∗	0.54∗∗∗	0.49∗∗∗	0.56∗∗∗	0.45∗∗∗
	[0.743 - 0.993]	[0.438 - 0.731]	[0.276 - 0.514]	[0.546 - 0.773]	[0.200 - 0.433]	[0.207 - 0.439]	[0.328 - 0.585]	[0.279 - 0.495]	[0.440 - 0.649]	[0.395 - 0.583]	[0.444 - 0.676]	[0.273 - 0.621]
Time (reference = 2 months after endline)												
6 months after endline	0.70∗∗∗	−0.07	−0.08	0.23∗∗∗	−0.18∗	0.37∗∗∗	−0.58∗∗∗	−0.35∗∗∗	0.04	0.07	−0.11	0.98∗∗∗
	[0.561 - 0.843]	[-0.241 - 0.105]	[-0.224 - 0.056]	[0.100 - 0.368]	[-0.317 to −0.041]	[0.238 - 0.511]	[-0.734 to −0.431]	[-0.477 to −0.230]	[-0.082 - 0.156]	[-0.038 - 0.179]	[-0.251 - 0.022]	[0.798 - 1.156]
12 months after endline	0.92∗∗∗	0.25∗∗	−0.15∗	0.23∗∗	−1.04∗∗∗	−0.45∗∗∗	−0.22∗∗	−0.04	0.12	0.03	0.04	1.37∗∗∗
	[0.774 - 1.069]	[0.069 - 0.430]	[-0.297 to −0.005]	[0.091 - 0.371]	[-1.188 to −0.900]	[-0.593 to −0.308]	[-0.378 to −0.062]	[-0.165 - 0.094]	[-0.003 - 0.246]	[-0.080 - 0.147]	[-0.099 - 0.186]	[1.182 - 1.558]
Treatment ∗ Time												
Treatment ∗ 6 months after endline	0.10	0.42∗∗∗	0.21∗	0.03	0.32∗∗∗	−0.01	0.13	0.18∗	−0.12	−0.15∗	−0.28∗∗	0.09
	[-0.078 - 0.271]	[0.206 - 0.635]	[0.037 - 0.384]	[-0.134 - 0.199]	[0.152 - 0.493]	[-0.179 - 0.159]	[-0.059 - 0.317]	[0.024 - 0.331]	[-0.263 - 0.032]	[-0.281 to −0.013]	[-0.453 to −0.114]	[-0.128 - 0.315]
Treatment∗ 12 months after endline	−0.21∗	0.32∗∗	0.23∗	0.07	0.33∗∗∗	0.29∗∗	−0.07	−0.06	−0.13	−0.16∗	−0.48∗∗∗	−0.35∗∗
	[-0.391 to −0.026]	[0.097 - 0.543]	[0.046 - 0.406]	[-0.103 - 0.243]	[0.153 - 0.508]	[0.114 - 0.466]	[-0.268 - 0.123]	[-0.222 - 0.097]	[-0.282 - 0.024]	[-0.296 to −0.017]	[-0.655 to −0.302]	[-0.580 to −0.117]

Note: Estimates are from random-effects panel regressions using the propensity-score–matched sample. Models include household-level matching weights and adjust for screening covariates used for matching (per capita income, household size, respondent age, Venezuelan nationality status, prior rent delays, eligibility score, and neighborhood). Time is measured as months since endline (2, 6, and 12 months). The reference category for time is two months after endline. Robust standard errors clustered at the household level are used. 95% confidence intervals are reported in brackets. ∗p < 0.05, ∗∗p < 0.01, ∗∗∗p < 0.001. All standardized beta coefficients statistically significant at p < 0.001 are also significant at p < 0.0045, the alpha level after applying a Bonferroni correction to account for multiple comparisons.

Generally, treatment × time coefficients were smaller and not statistically significant, suggesting that the sizable initial treatment advantage was largely maintained rather than widening or diminishing over the follow-up period. A few exceptions were observed. For example, treatment households demonstrated modest but significant additional improvements in peace of mind at 6 months post-endline (B = 0.42; 95% CI[0.206 - 0.635]) and at 12 months post-endline (B = 0.32; 95% CI[0.097 - 0.543]) relative to controls; similar relative improvements were observed for self-efficacy at 6 months post-endline (B = 0.32; 95% CI[0.152 - 0.493] and at 12 months post-endline (B = 0.33; 95% CI[0. 0.153 - 0.508]). In contrast, while treatment households exhibited greater acculturation 2 months after endline, this effect attenuated at 6 and 12 months post endline. Similar attenuation was observed with treatment effects on income at 12 months post endline.

**Fig. 2:**
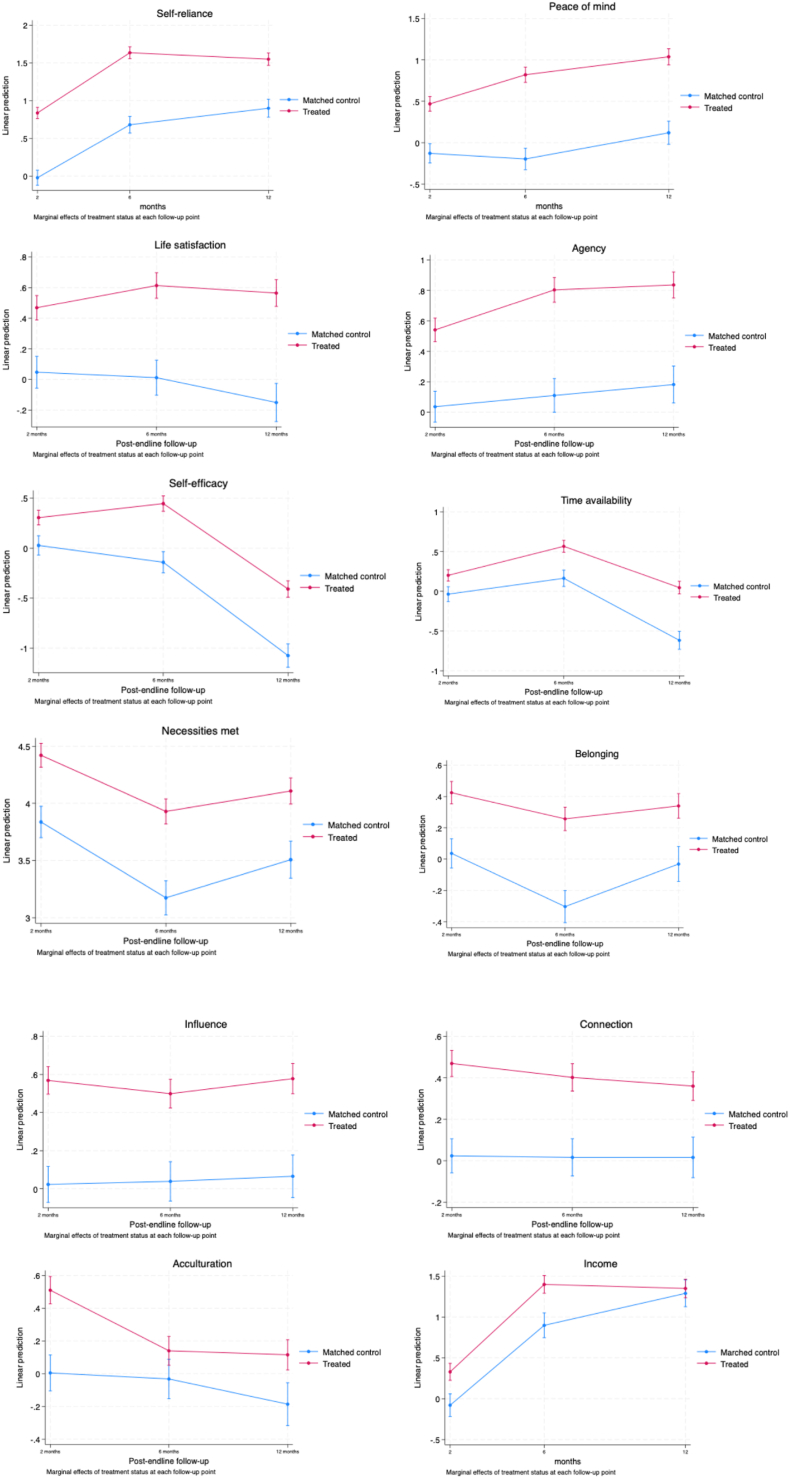
Marginal effects of treatment status at each follow-up point, random effects panel regression models.

Marginal effects of the intervention at each follow-up point are shown in Fig. 2 and highlight two outcomes that declined between 6 and 12 months post-endline: self-efficacy and time availability. However, decreases in these two outcomes were observed across both treatment and matched control groups, with the treatment group still exhibiting statistically greater values at both time points as compared to matched control. Overall, findings indicate that the intervention produced meaningful gains that persisted through one year post-intervention, with no systematic convergence between groups on key indicators of well-being and self-reliance. Per capita household income in the last month proved the one exception, whereby treatment effects observed at six months post-endline had disappeared by the 12-month mark.

## 4 Discussion

This study provides evidence that short-term rental assistance is associated with significant and sustained improvements in household-level self-reliance and multiple dimensions of well-being among forcibly displaced populations. Across nearly all outcomes assessed, treatment households outperformed matched controls at two, six, and twelve months post-endline, with large effect sizes persisting well beyond the end of rental coverage. In addition to improvements in self-reliance, we observed meaningful gains in peace of mind, life satisfaction, agency, and other indicators aligned with reduced scarcity mindset and increased psychological stability. Although the size of treatment effects declined slightly over time for a few outcomes (particularly those tied to neighborhood-level connection and resources) and disappeared altogether by 12 months after endline for income, treatment effects for the majority of outcomes remained statistically significant through the twelve-month post-implementation follow-up. Random-effects models confirmed that these treatment–control gaps did not significantly narrow over time for most outcomes, suggesting that the intervention's early benefits were largely maintained. Taken together, these findings suggest that providing housing support to displaced households may meaningfully reduce psychosocial burden and create lasting conditions for economic and emotional recovery.

Venezuelan households in Colombia face profound and persistent depravation with respect to basic needs and consumption (Wirtz et al., 2024). Research integrating poverty and scarcity lenses suggests that such chronically insecure households—as compared to those facing incidental or conditional scarcity, for example—are often unable to restore resources without intervention (Blocker et al., 2023). The present housing intervention produced significant improvements in household self-reliance and in a range of well-being outcomes, including peace of mind, agency, and time availability, all of which may serve as a proxy for the scarcity mindset. Findings thus lend empirical support to existing conceptual models positing that secure and adequate housing can improve psychosocial outcomes by reducing stress and enabling individuals to engage in longer-term planning and resource-building (Swope & Hernández, 2019).

However, the precise mechanisms through which housing assistance promotes recovery remain unclear—particularly for displaced populations, who may continue to encounter structural and social barriers even when their housing and other basic needs are met. Future research should further investigate the pathways linking housing, cognitive scarcity, self-reliance, income, and well-being. Importantly, treatment effects for per capita household income were no longer observed at 12 months after endline, despite sustained treatment effects for other measures of well-being. This finding suggests that while housing support may partially impact psychosocial outcomes through increased capacity to invest in longer-term financial well-being, there are other mechanisms of change playing a role as well. Clarifying these mechanisms will be essential for designing future programs that integrate housing assistance with complementary interventions to accelerate recovery and build sustainable well-being in displacement-affected populations.

For example, future research should explore the role of perceived and actual safety as a mediating factor between housing support and well-being, as a key element of the rental-assistance intervention was the requirement that participants secure a *safe* housing option before receiving rental coverage. A prior evaluation of this housing intervention found substantial improvements in participants' perceptions of safety (Stark et al., 2025), suggesting that stability and protection may have played a critical role in altering psychosocial conditions. In the broader literature, perceptions of lower safety, whether at home or in one's neighborhood, have been linked with elevated stress, depressive symptoms, and reduced psychological functioning even after controlling for objective indicators of crime (Holding et al., 2020; Robinette et al., 2021). When individuals feel unsafe, cognitive and emotional resources may be consumed by vigilance, fear, and threat mitigation, reducing capacity for agency, self-efficacy and future-oriented investment. Conversely, improved safety can free mental bandwidth, strengthen self-regulation, and enable households to engage in behaviors aligned with longer-term goals. In displacement-affected contexts, where protection risks, unstable tenancy, and neighborhood insecurity are common, ensuring safe shelter may function not simply as a material benefit, but as a psychosocial stimulus for recovery and self-reliance (Ziersch et al., 2024). Evidence from this study suggests that housing assistance may serve as a valuable intervention for especially vulnerable subgroups of displaced households, such as female-headed families, those with protection concerns, or those living in high-risk shelter conditions.

Not only do our findings suggest that housing may be an impactful intervention to improve self-reliance and mental well-being among vulnerable subgroups of displaced households, they also provide evidence for what may be a cost-effective intervention. In this study, the nine-month rental assistance program led to substantial and durable gains across nearly all outcomes, with treatment households exhibiting significantly higher self-reliance even 12 months after the end of the intervention. This indicates a sustained return on investment and suggests that stabilized housing conditions may generate benefits long after support concludes. In contrast, the small number of studies that evaluate livelihoods and skills-training interventions for refugees — though widely implemented — show mixed impacts on sustained gains in income, employment, and mental health, while demonstrating expensive programmatic costs (Kaumba, 2024; Yan et al., 2025). Future research should compare the cost -effectiveness of housing assistance to these other commonly funded interventions in humanitarian contexts. Measurements may include the relative cost per unit increase in self-reliance and mental health scores, as well as long-term economic trajectories of study participants.

## 5 Limitations

This study has several limitations. First, because participation in the rental assistance program was determined through a needs-based vulnerability screening process rather than random assignment, the analysis relies on propensity score matching to construct a comparable control group. While matching substantially reduced observable differences between treated and untreated households, causal interpretation depends on the assumption that the variables included in the propensity score model captured the most relevant determinants of both program participation and subsequent outcomes. Unobserved factors influencing program prioritization or household trajectories may still introduce bias. Second, baseline outcome measures were not collected for the control group prior to the start of the intervention, limiting our ability to directly assess pre-intervention equivalence in self-reliance and psychosocial outcomes. Instead, comparability between groups is inferred through balance in observed baseline characteristics. Third, differential attrition across follow-up waves may affect the representativeness of the longitudinal sample. Although we examine patterns of loss to follow-up and do not identify any meaningful bias, these factors should be considered when interpreting the findings. Taken together, the results should therefore be interpreted as evidence from a quasi-experimental evaluation rather than a fully randomized causal design.

## 6 Conclusion

This study provides evidence that short-term rental assistance may generate impactful and sustained improvements in self-reliance and psychosocial well-being, as well as other indicators of success, for female-headed households among Venezuelan refugees in Colombia. By alleviating housing insecurity, the intervention reduces the scarcity mindset and enables households to stabilize, plan ahead, and invest in long-term goals. This is an especially notable contribution to the literature given the relatively few studies that examine housing as an intervention for displaced households, and many studies that find mixed results for livelihoods interventions. Rental assistance may offer a highly efficient pathway to economic independence, psychosocial wellbeing, and self-reliance. Taken together, the findings from this study may help to guide policy and program toward interventions that produce sustainable and equitable improvements for highly vulnerable displaced persons.

## Ethics statement

The data analyzed in this study were originally collected by the implementing organization as part of its routine administrative and program monitoring procedures, rather than for research purposes. As such, formal ethics approval was not sought at the time of data collection. Prior to data collection, participants were informed about the purpose of the data collection activities, the procedures involved, any potential risks and benefits, and their right to decline participation or withdraw at any time without penalty. The present study involved secondary analysis of de-identified administrative/program monitoring data and was deemed exempt from review by the Institutional Review Board at Washington University in St. Louis.

## Financial disclosure statement

This research was funded by Washington University in St Louis (grant numbers PJ000032066 and PJ000032416).

## CRediT authorship contribution statement

**Ilana Seff:** Conceptualization, Data curation, Formal analysis, Funding acquisition, Methodology, Writing – original draft, Writing – review & editing. **Juan Pablo Franco:** Conceptualization, Data curation, Funding acquisition, Project administration, Writing – review & editing. **Arturo Harker Roa:** Conceptualization, Methodology, Writing – review & editing. **Deanna Barch:** Conceptualization, Funding acquisition, Writing – review & editing. **Kellie Leeson:** Conceptualization, Writing – review & editing. **Lindsay Stark:** Conceptualization, Funding acquisition, Methodology, Writing – review & editing.

## Declaration of competing interest

The authors declare that they have no known competing financial interests or personal relationships that could have appeared to influence the work reported in this paper.

## Data Availability

Data will be made available on request.
