# Supplementary material for: Evaluating the relationship between rental assistance and self-reliance and well-being among displaced populations: A propensity score–matched analysis
Source: SSM Popul Health. 2026 Jul 9;35:101948. doi: 10.1016/j.ssmph.2026.101948 (PMC13382798; doi:10.1016/j.ssmph.2026.101948)
Supplement: Multimedia component 1 [file mmc1.docx]

Supplementary File S1

Table S1. Correlation matrix of outcomes at two months post-endline

|  | SRI | Peace of mind | Life satisfaction | Agency | Self-efficacy | Time availability | Necessities met | Belonging | Influence | Connection | Acculturation | Per capita household income |
| --- | --- | --- | --- | --- | --- | --- | --- | --- | --- | --- | --- | --- |
|  |  |  |  |  |  |  |  |  |  |  |  |  |
| SRI | 1.000 |  |  |  |  |  |  |  |  |  |  |  |
|  |  |  |  |  |  |  |  |  |  |  |  |  |
|  |  |  |  |  |  |  |  |  |  |  |  |  |
| Peace of mind | 0.339 | 1.000 |  |  |  |  |  |  |  |  |  |  |
|  | 0.000 |  |  |  |  |  |  |  |  |  |  |  |
|  |  |  |  |  |  |  |  |  |  |  |  |  |
| Life satisfaction | 0.390 | 0.448 | 1.000 |  |  |  |  |  |  |  |  |  |
|  | 0.000 | 0.000 |  |  |  |  |  |  |  |  |  |  |
|  |  |  |  |  |  |  |  |  |  |  |  |  |
| Agency | 0.332 | 0.315 | 0.352 | 1.000 |  |  |  |  |  |  |  |  |
|  | 0.000 | 0.000 | 0.000 |  |  |  |  |  |  |  |  |  |
|  |  |  |  |  |  |  |  |  |  |  |  |  |
| Self-efficacy | 0.297 | 0.341 | 0.245 | 0.354 | 1.000 |  |  |  |  |  |  |  |
|  | 0.000 | 0.000 | 0.000 | 0.000 |  |  |  |  |  |  |  |  |
|  |  |  |  |  |  |  |  |  |  |  |  |  |
| Time availability | 0.134 | 0.261 | 0.121 | 0.117 | 0.147 | 1.000 |  |  |  |  |  |  |
|  | 0.000 | 0.000 | 0.001 | 0.002 | 0.000 |  |  |  |  |  |  |  |
|  |  |  |  |  |  |  |  |  |  |  |  |  |
| Necessities met | 0.283 | 0.311 | 0.346 | 0.358 | 0.335 | 0.143 | 1.000 |  |  |  |  |  |
|  | 0.000 | 0.000 | 0.000 | 0.000 | 0.000 | 0.000 |  |  |  |  |  |  |
|  |  |  |  |  |  |  |  |  |  |  |  |  |
| Belonging | 0.352 | 0.221 | 0.337 | 0.403 | 0.340 | 0.091 | 0.559 | 1.000 |  |  |  |  |
|  | 0.000 | 0.000 | 0.000 | 0.000 | 0.000 | 0.016 | 0.000 |  |  |  |  |  |
|  |  |  |  |  |  |  |  |  |  |  |  |  |
| Influence | 0.355 | 0.252 | 0.327 | 0.389 | 0.330 | 0.099 | 0.476 | 0.625 | 1.000 |  |  |  |
|  | 0.000 | 0.000 | 0.000 | 0.000 | 0.000 | 0.009 | 0.000 | 0.000 |  |  |  |  |
|  |  |  |  |  |  |  |  |  |  |  |  |  |
| Connection | 0.372 | 0.275 | 0.338 | 0.472 | 0.400 | 0.115 | 0.517 | 0.726 | 0.689 | 1.000 |  |  |
|  | 0.000 | 0.000 | 0.000 | 0.000 | 0.000 | 0.002 | 0.000 | 0.000 | 0.000 |  |  |  |
|  |  |  |  |  |  |  |  |  |  |  |  |  |
| Acculturation | 0.234 | 0.111 | 0.172 | 0.190 | 0.219 | 0.050 | 0.210 | 0.322 | 0.272 | 0.288 | 1.000 |  |
|  | 0.000 | 0.003 | 0.000 | 0.000 | 0.000 | 0.187 | 0.000 | 0.000 | 0.000 | 0.000 |  |  |
|  |  |  |  |  |  |  |  |  |  |  |  |  |
| Per capita household income | 0.339 | 0.166 | 0.241 | 0.192 | 0.153 | -0.106 | 0.114 | 0.123 | 0.145 | 0.186 | 0.164 | 1.000 |
|  | 0.000 | 0.000 | 0.000 | 0.000 | 0.000 | 0.005 | 0.003 | 0.001 | 0.000 | 0.000 | 0.000 |  |

Note: Cells present correlations (r) and P values.
