# Supplementary material for: Evaluating the relationship between rental assistance and self-reliance and well-being among displaced populations: A propensity score–matched analysis
Source: SSM Popul Health. 2026 Jul 9;35:101948. doi: 10.1016/j.ssmph.2026.101948 (PMC13382798; doi:10.1016/j.ssmph.2026.101948)
Supplement: Multimedia component 2 [file mmc2.docx]

Supplementary File S2
