# Supplementary figures and images for: Evaluating the relationship between rental assistance and self-reliance and well-being among displaced populations: A propensity score–matched analysis
Source: SSM Popul Health. 2026 Jul 9;35:101948. doi: 10.1016/j.ssmph.2026.101948 (PMC13382798; doi:10.1016/j.ssmph.2026.101948)

Supplementary Figure S3

Pstest output for propensity score matching


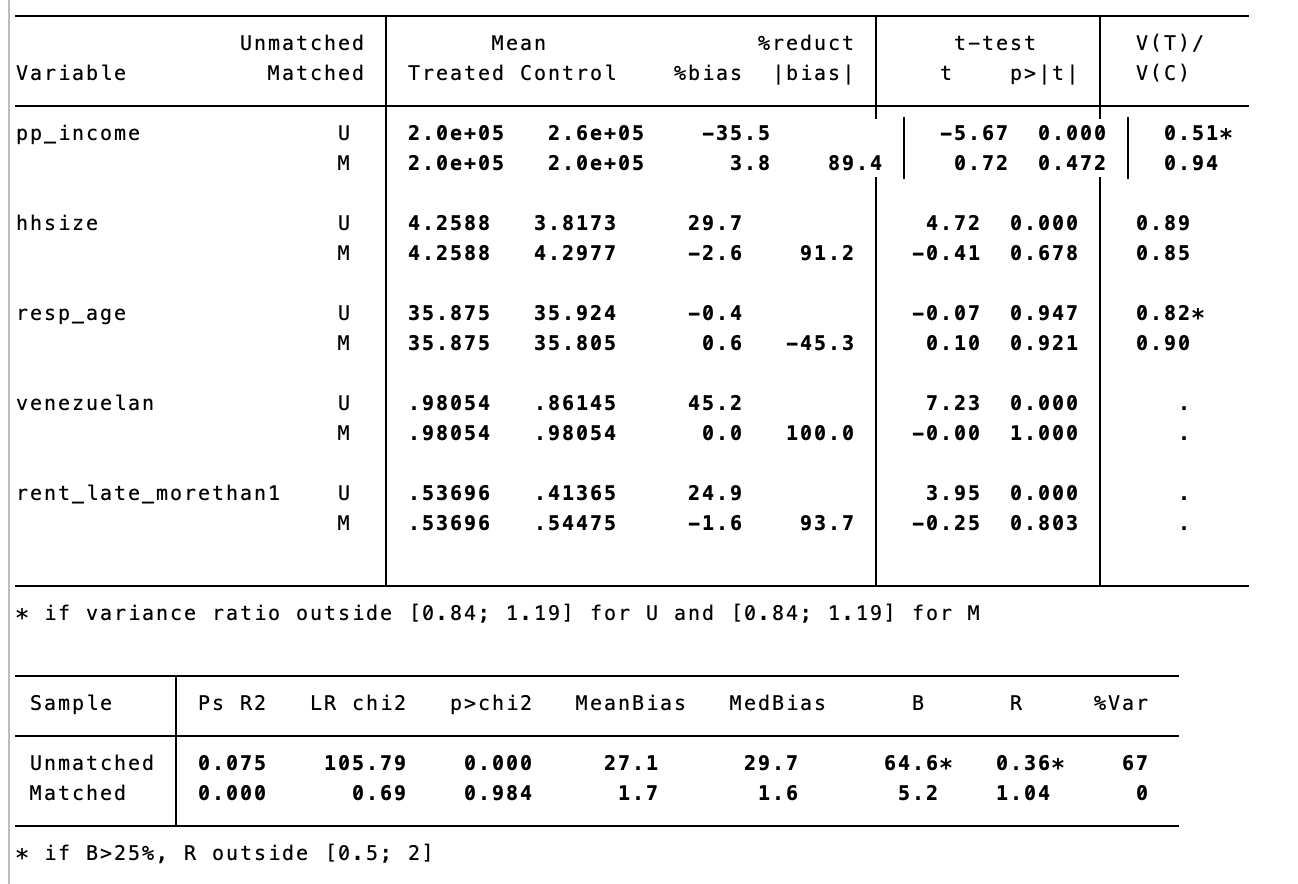

Supplement: Multimedia component 3 [file mmc3.docx]
